# Effect of ramosetron, a 5-HT_3_ receptor antagonist on the severity of seizures and memory impairment in electrical amygdala kindled rats

**DOI:** 10.1186/s12576-022-00825-5

**Published:** 2022-01-16

**Authors:** Zeynab Sayahi, Alireza Komaki, Masoud Saidi Jam, Seyed Asaad Karimi, Safoura Raoufi, Parastoo Mardani, Marzieh Naderishahab, Abdolrahman Sarihi, Javad Mirnajafi-Zadeh

**Affiliations:** 1 Neurophysiology Research Center, Hamadan University of Medical Sciences, Shahid Fahmideh Street, 6517838736 Hamadan, Iran; 2 Department of Neuroscience, School of Sciences and Advanced Technology in Medicine, Hamadan University of Medical Sciences, Hamadan, Iran; 3 Research Center for Molecular Medicine, Hamadan University of Medical Sciences, Hamadan, Iran; 4 Department of Biology, Faculty of Sciences, Payame Noor University, Sanandaj, Iran; 5 Department of Physiology, Faculty of Medical Sciences, Tarbiat Modares University, 1411713116 Tehran, Iran

**Keywords:** Seizure, 5-HT_3_ receptor, Entorhinal cortex, Amygdala kindling, Ramosetron

## Abstract

The entorhinal cortex (EC) plays a pivotal role in epileptogenesis and seizures. EC expresses high density of serotonergic receptors, especially 5-HT_3_ receptors. Cognitive impairment is common among people with epilepsy. The present study investigated the role of 5-HT_3_ receptor on the severity of seizures and learning and memory impairment by electrical kindling of amygdala in rats. The amygdala kindling was conducted in a chronic kindling manner in male Wistar rats. In fully kindled animals, ramosetron (as a potent and selective 5-HT_3_ receptor antagonist) was microinjected unilaterally (ad doses of 1, 10 or 100 µg/0.5 µl) into the EC 5 min before the novel object recognition (NOR) and Y-maze tests or kindling stimulations. Applying ramosetron at the concentration of 100 μg/0.5 µl (but not at 1 and 10 µg/0.5 µl) reduced afterdischarge (AD) duration and increased stage 4 latency in the kindled rats. Moreover, the obtained data from the NOR test showed that treatment by ramosetron (10 and 100 µg/0.5 µl) increased the discrimination index in the fully kindled animals. Microinjection of ramosetron (10 and 100 µg/0.5 µl) in fully kindled animals reversed the kindling induced changes in the percentage of spontaneous alternation in Y-maze task. The findings demonstrated an anticonvulsant role for a selective 5-HT_3_ receptor antagonist microinjected into the EC, therefore, suggesting an excitatory role for the EC 5-HT_3_ receptors in the amygdala kindling model of epilepsy. This anticonvulsive effect was accompanied with a restoring effect on cognitive behavior in NOR and Y-maze tests.

## Background

Epilepsy is a chronic neurological disorder, with a prevalence of about 1%, which is characterized by the recurrent appearance of spontaneous seizures due to pathological hyperexcitability and sudden abnormal discharge of neurons in the neuronal network [1]. The most common epileptic syndrome in adults is temporal lobe epilepsy (TLE). It is the most drug-resistant type of adult focal epilepsy. One of the most commonly used animal models of TLE is the kindling model of epilepsy [2]. The epileptic focus in TLE patients or TLE-like animal models often resides in one of the temporal lobe structures, notably the hippocampus, the amygdala, or in both regions.

The amygdala is among the most vulnerable areas to kindling [1, 3]. Thus, amygdala kindling is a widespread experimental model for TLE with complex partial and/or secondarily generalized seizures [4]. Since amygdala plays a key role in cognitive and emotional functions [5], the amygdala dysfunction in TLE is essential not only for its role in the generation of seizures, but also for its role in the psychological disorders that are often associated with epilepsy [3, 6]. Using this model, evaluating the possible involvement and anticonvulsant effects of major neurotransmitter systems in nervous system can be done. The entorhinal cortex (EC) located in the anterior parahippocampal gyrus is a major source of inputs to the hippocampus. In addition, the cingulate cortex, temporal lobe cortex, amygdala, orbital cortex, and olfactory bulb all have inputs to the hippocampus via the EC [7]. Therefore, EC is a major relay for propagating the seizure activity from hippocampus to other brain areas and serves as the major interface between the hippocampus and sensory cortices [8]. Hence, hippocampal memory function depends on an intact EC [9].

Bagdy et al. have reported a relationship between serotonin (5-hydroxytryptamine or 5-HT) and epilepsy [3]. In addition, much evidence has suggested that changes in 5-HT-mediated serotonergic neurotransmission may be the main mechanism for the onset and progression of epilepsy [1, 10]. Serotonin is a biogenic monoamine that acts as a classical neurotransmitter and mediates numerous physiological processes in the central nervous systems (CNS) [2, 3]. 5-HT receptors are a group of G protein-coupled receptors and ligand-gated ion channels which can be classified into seven distinct families (5-HT_1_ to 5-HT_7_) according to their structural diversity and mode of action [4]. Among the seven known classes of receptors for 5-HT, the 5-HT_3_ receptor is unique as being a ligand-gated ion channel [5]. 5-HT_3_ receptors are located pre- and post-synaptically in both the peripheral (PNS) and CNS [3, 6]. 5-HT_3_ receptors in the CNS may be active in a variety of functions including emesis, cognition as well as anxiety. 5-HT_3_ receptor activation enhances the release of a variety of neurotransmitters including dopamine, cholecystokinin and GABA [7]. They are located in many brain areas including cortex, hippocampus, nucleus accumbens, and EC [7]. The EC expresses high density of serotonergic receptors including 5-HT_1A_, 5-HT_1D_, 5-HT_1E_, 5-HT_2A_, 5-HT_3_ and 5-HT_6_ [11]. Radioligand binding studies have been shown the highest density of 5-HT_3_ in cortical area including EC [9]. The EC is essential not only for many physiological and pathological condition, but also takes part in seizure generation and propagation in TLE [3].

The role of 5-HT receptors is related to intrinsic neuronal and synaptic excitability levels [10]. Studies have shown that activation of the 5-HT_3_ receptor through SR57227 is related to PTZ-induced seizures and possibly related to hippocampal GABA activity [12]. Notable, among the 5-HT receptor family, only 5-HT_3_ receptors are ligand gated ion channels that can directly or indirectly act by changing cell ion conductance or concentration leading to neuronal depolarization. It is not surprising, then, that any major shift in 5-HT receptors in the body is related to the induction of epilepsy [13].

In previous studies, researchers have attempted to examine the relationship between the 5-HT_3_ receptor and seizure. For example, Wada et al. showed that intracerebroventricular administration of m-CPBG, a 5-HT_3_ receptor agonist, increased the seizure duration in fully kindled rats and facilitated the developmental seizure process and, therefore, suggesting an excitatory role for 5-HT_3_ receptors in the amygdala kindling model of epilepsy [14]. In addition, it has been suggested that activation of 5-HT_3_ receptor by SR 57227, significantly prolonged seizure latency and decreased seizure score in pentylenetetrazole-induced seizures in mice [1]. Furthermore, Gholipour and colleagues demonstrated that i.p. injection of SR57227 hydrochloride increased the pentylenetetrazole-induced seizure threshold in mice and showed that selective antagonism at the 5-HT_3_ receptor yields proconvulsive effects [15].

In vitro experiments have shown that blocking the 5-HT_3_ receptor in mice causes significantly delayed epileptic seizures induced in vivo, and may even stop them completely [10]. The anti-epileptic activity of selective 5-HT reuptake inhibitor has also been reported [16]. Ondansetron is a highly selective 5-HT_3_ receptor antagonist, that has proven effective in treating several diseases such as anxiety, itching, refractory chronic diarrhea, irritable bowel syndrome, and epilepsy [17], and researchers have demonstrated its anticonvulsant potential in experimental seizure models as well [18, 19]. Other experiments have shown that adding the inhibitor causes the seizure rate of mice to increase [20]. According to shock experiments, the protective effect of Ondansetron may be due to the cation influx of change Na^+^, Ca^2+^, or K^+^ leading to neuronal depolarization inhibition [18]. In a mouse model Ondansetron showed anticonvulsant effect against epileptic seizures in accordance with results obtained by Mohanty and Balakrishnan et al. [19]. However, the precise role of EC’s 5-HT_3_ receptors in TLE has not been completely determined.

Amygdala is one of the principal targets of the EC. Therefore, in the present study in an effort to better understand the relationship between epilepsy and the 5-HT_3_ receptors in EC, the effects of 5-HT_3_ receptor antagonist—ramosetron—on the severity of seizures was investigated. Several lines of evidences have shown that impairment in cognitive functions is related to the seizure focus, especially the EC which has an important role in various forms of memory [21, 22]. Considering the role of these receptors in cognitive behaviors [23] and the role of EC in working memory [24] and novel object recognition test [25], the probable effect of ramosetron on electrical amygdala kindling-induced memory impairment was also evaluated.

## Materials and methods

### Animal

Male Wistar rats weighing between 290 and 350 g were used in this study**.** Kindling in rodents is a use full tool to model human limbic epilepsy. The animal strain, species, and age can have a profound influence on measures of seizure susceptibility and epilepsy [26]. Outbred strains of mice (e.g., Swiss, NMRI or CD-1) or rats (e.g., Wistar or Sprague–Dawley (SD)) have been widely used in models of seizures or epilepsy, but such outbred strains can increase seizure variability with a high intrastrain phenotypic variation due to genetic heterogeneity [27, 28]. Seizure-related behaviors are also age-dependent. Compared with P18, P28, and adult rats, P10 animals injected with PTZ have shorter latency to generalized tonic–clonic seizures [29]. In our work, the animals were stimulated according to the chronic kindling protocol (1 stimulation/day). For generating seizure or epilepsy models in rats, outbred strains such as Wistar or SD are often used. Although the Wistar rats are less sensitive to status epilepticus induction than SD rats, but also Wistar rats are susceptible to convulsive seizures [26]. In addition, in our work, we used Wistar rats. The animals were kept in cages with 2–3 rats in each before surgery or one after surgery with ad libitum access to food and water. They were housed at controlled temperature (23 ± 1 °C) and 12-h light–dark cycle (lights on between 7:00 AM and 7:00 PM). Experiments were carried out between 14:00 and 17:00. All experimental and animal care procedures were performed according to international guidelines for the use of laboratory animals and approved by “Hamadan University of Medical Sciences Ethical Committee for Animal Research” which is in line with the “NIH Guide for the Care and Use of Laboratory Animals”. All Efforts were made to minimize both the number of animals used and their suffering.

### Surgical procedures

The rats were deeply anesthetized by a ketamine/xylazine mixture (100/10 mg/kg, i.p, respectively) and fixed in a stereotaxic frame with their skulls exposed. Bipolar stimulating and monopolar recording electrodes (twisted into a tripolar electrode) were implanted into the right basolateral amygdala (− 2.5 mm posterior, 4.8 mm lateral to the bregma, and 8.5 mm below the skull). For chemical microinjection, a 22-gauge guide cannula was implanted into the EC (coordinates: − 6.7 mm posterior, 4.2 lateral to the bregma and 8.8 mm below dura according to Paxinos and Watson atlas) [30]. The electrodes (125 μm in diameter; A.M. Systems Inc., USA) were teflon coated and isolated in all length, except for their tips. A monopolar electrode connected to a stainless steel screw was also positioned in the skull above the occipital area as a reference and/or ground electrode. All electrodes were connected to metal pins and put in a small plastic socket. The socket was fixed to the skull using dental acrylic. Both electrode and guide cannula were implanted ipsilateral.

### Kindling procedure

**Fig. 1:**
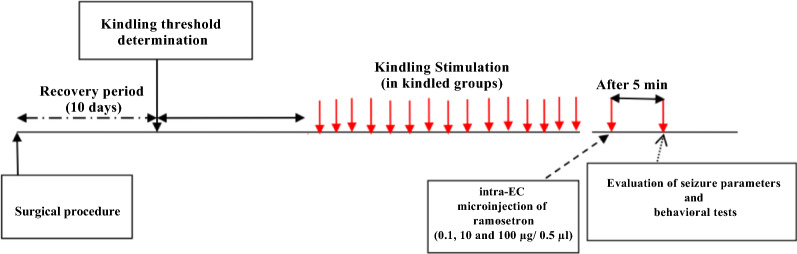
Time line diagram showing the experimental protocol used in kindled groups

Ten days after surgery (Fig. 1), the afterdischarge (AD) threshold was determined in basolateral amygdala via a train of monophasic square waves (1 ms pulse duration at the frequency of 50 Hz for 3 s). Briefly, the stimulating currents were initially delivered at 30 μA and then the stimulus intensity was increased in steps of 10 μA at 10 min intervals until ADs were recorded for at least 8 s [31]. Animals were stimulated according to the chronic kindling protocol (1 stimulation/day). All epileptiform ADs were continuously recorded from the basolateral amygdala using a PC-based electromodule system (D3107; ScienceBeam Co., Tehran, Iran). The behavioral seizure severity was rated according to Racine’s scores [32]: stage 0, no convulsion; stage 1, facial automatism; stage 2, head nodding; stage 3, unilateral forelimb clonus; stage 4, bilateral forelimb clonus; and stage 5, rearing, falling, and generalized convulsions. The animals were considered as fully kindled when they exhibited stage 5 seizure in three consecutive days. The animals achieved a stage 5 seizure after 10–15 days. Afterdischarge duration (ADD), latency to stage 4 seizure (S4L; as an index of latency to start of generalized seizures), stage 5 seizure duration (S5D), total seizure duration (SD) and the behavioral seizure stage were calculated following each kindling stimulation.

### Drug preparation and microinjection

Ramosetron (abcam, UK), as a potent and selective 5-HT_3_ receptor antagonist, was dissolved in DMSO. Drug was infused via a 30-gauge cannula, which was 1 mm below the tip of 22-gauge cannula. In fully kindled rats, ramosetron (1, 10, 100 µg/0.5 µl) was microinjected into the EC and 5 min later the animals were stimulated at AD threshold. In each case, 24 h prior to the experiment, the animals received DMSO and were stimulated in the same way, where the results recorded as baseline values.

### Open field test (locomotion)

The open-field test (OFT) measures locomotor activity, motor impairment, and anxiety in rodents. OFT was first developed to measure emotions in rats [33]. The rats were transferred to the testing room in their home cages and allowed to habituate through 30 min prior to testing. The apparatus was made of a black round arena (48 × 41.5 × 36 cm) elevated 60 cm above the floor. The floor can be seen with a grid of 16 squares. Using a video camera, the paths traveled by animals were recorded within 10 min. The numbers of squares crossed using four paws were calculated. The OFT box was washed using a 70% alcohol solution before placing other rats for preventing olfactory perception by other animals [34, 35].

### Novel object recognition test (NOR)

As previously reported in our previous work, the NOR test is referred as “pure” recognition memory test [31]. Briefly, rats were allowed 10 min of habituation sessions in an evenly lit plastic container (38 × 48 × 42 cm) with no objects. The following day, 12 h later, the rats were given time to explore two similar objects. The objects were located at the same point at all times, for a 5 min training session. Fifteen minutes after the training session, memory retention was assessed in a test session. During a 3 min test session, familiar and novel objects were placed in positions identical to where the objects were in the training phase. To avoid animals’ natural preference of one location or another during the test phase, the location of novel objects were changed randomly. The objects were composed of the same materials and had a similar size to the training objects, but with different shapes. The container and objects were cleaned with 70% alcohol and air-dried after each animal change. Exploration time was quantified by measuring the time that animals sniffed or touched the object with their nose being recorded with a camera. Then, a discrimination index was obtained as ratio of time spent for exploring each object to the total time spent for exploring both objects multiplied by 100 [36].

### Y-maze task

Y-maze (Y-shaped maze) is a behavioral test for measuring the willingness of rodents to explore new environments. Rodents typically prefer to investigate a new arm of the maze rather than returning to one that was previously visited. The Y-maze is a hippocampal dependent–spatial working memory task that requires rats to use external maze cues to navigate the identical internal arms. The Y-maze was chosen to reduce habituation time, provide a measure of spatial working memory, and limit stressful confounds, such as food deprivation (radial arm maze) or forced swimming (water maze). The apparatus consisted of a black plastic maze with three arms (50 cm long, 32 cm high, and 16 cm wide) that were intersected at 120^◦^. A rat was placed at the end of one arm and allowed to move freely through the maze for 8 min without reinforcements, such as food and water. Entries into all arms were noted (4 paws had to be inside the arm for a valid entry) and a spontaneous alternation was counted if an animal entered three different arms consecutively. The percentage of spontaneous alternation was calculated according to following formula [25]:

$$\left[ {\left( {\text{number of alternations}} \right)/\left( {{\text{total number of arm entries }} - { 2}} \right)} \right] \times {1}00.$$

### Experimental design

Animals were randomly assigned to following groups as control, kindled, kindled + vehicle, kindled + ramosetron at the doses of 1,10 and 100 µg/0.5 µl. After achieving the fully kindled state, the animals were stimulated 3–5 times and the averaged seizure parameters during these 3–5 days were used for data analysis. In kindled + vehicle group, fully kindled animals received intra-EC drug’s vehicle and then received kindling stimulations. In kindled + ramosetron groups, fully kindled animals received intra-EC ramosetron (and kindling stimulation were applied 5 min later). The seizure parameters were calculated and monitored after drug or vehicle injection and compared with their amounts measured before injections.

### Statistical analysis

Data expressed as mean ± Standard Error of the Mean (SEM) and analyzed by GraphPad Prism 8. The data were compared in different experimental groups by one-way analysis of variance (ANOVA) followed by Tukey's multiple comparison test. The probability level interpreted as statistically significant was *P* < 0.05.

## Results

There was no significant difference in kindling rate between the animals in different experimental groups. The mean number of stimulations (per day) to achieve fully kindled state was 17.16 ± 2.4 in kindled, 16.33 ± 1.3 in kindled + vehicle and 18.25 ± 1.31 in kindled + ramosetron rats. In addition, AD threshold showed no significant difference in these three experimental groups and was 54.78 ± 4.45 µA, 65.55 ± 5.46 µA and 54.5675 ± 2.56 µA in kindled, kindled + vehicle and kindled + ramosetron, respectively.

### Effect of ramosetron on seizure parameters of fully kindled animals

**Fig. 2:**
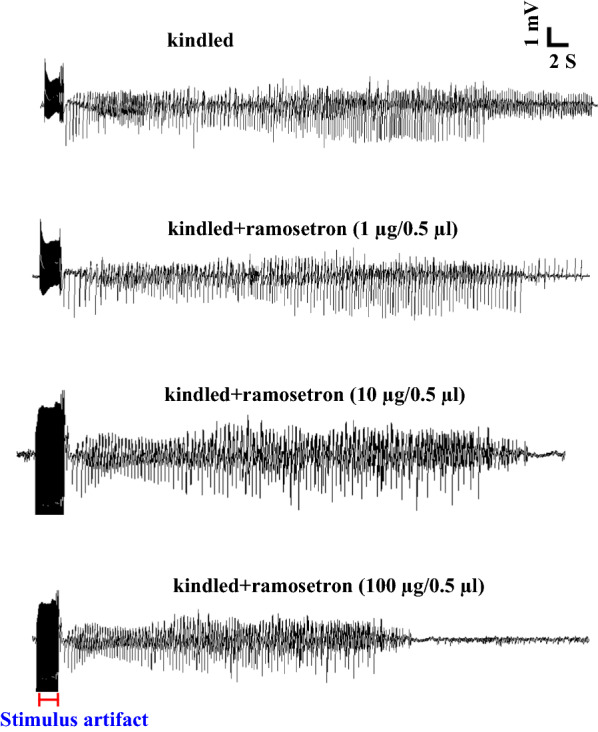
Electrographic examples of afterdischarge duration (ADD) taken from the amygdala in different experimental groups. The highest duration of ADs was observed in the kindled group compared with the others. Intra-EC microinjection of ramosetron at the dose of 100 µg/0.5 µl suppressed the ADD

**Fig. 3:**
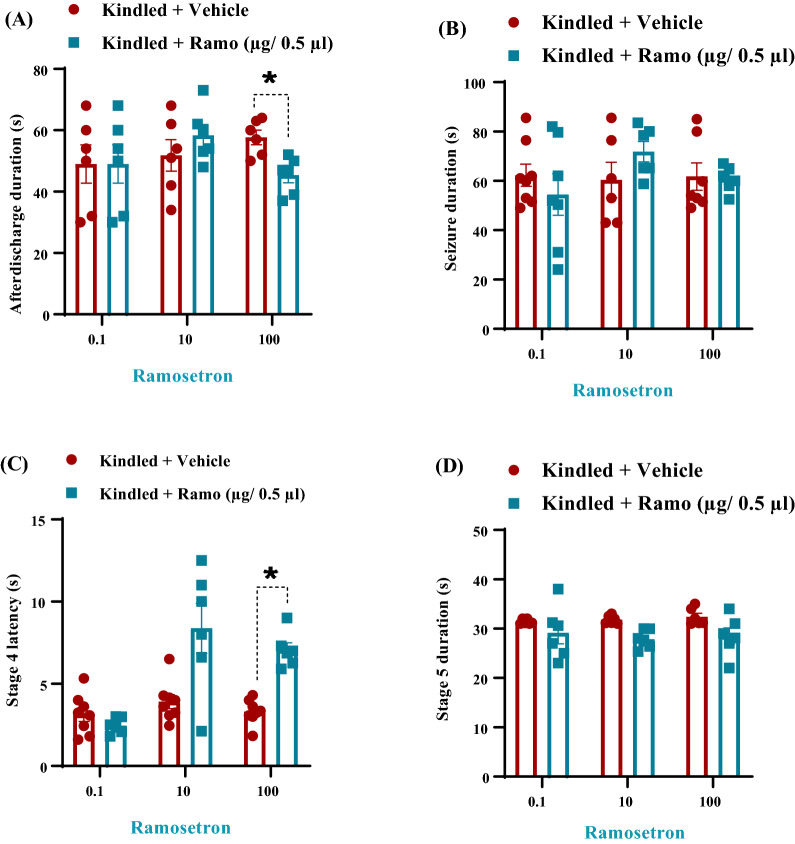
Effect of intra-EC microinjection of ramosetron (1, 10 and 100 µg/ 0.5 µl) on afterdischarge duration (**A**), seizure duration (**B**), stage 4 latency (**C**) and stage 5 duration (**D**) of amygdala-kindled rats. Data are expressed as mean ± SEM. *P < 0.05

Amygdala is one of the principal targets of the EC. Therefore, in the present study in an effort to better understand the relationship between epilepsy and the 5-HT_3_ receptors in EC, the effects of 5-HT_3_ receptor antagonist—ramosetron—on the severity of seizures was investigated. In fully kindled animals, the analysis showed the anticonvulsive effect of ramosetron. Intra-EC microinjection of ramosetron at 100 µg/0.5 µl dose had inhibitory effect on electrophysiological (Fig. 2) and behavioral parameters of kindling. Applying ramosetron at the dose of 100 µg/0.5 µl, but not at 1 and 10 µg/0.5 µl, reduced ADD in kindled animals (*P* < 0.05, Fig. 3A). However, this agent at all three doses had no significant effect on seizure duration (*P* > 0.05; Fig. 3B).

To determine the role of 5-HT3 receptor in mediating the generalization rate of the seizure attacks, the stage 4 latency was examined. The results revealed that ramosetron at the dose of 100 µg/0.5 µl increased stage 4 latency (S4L) (*P* < 0.05, Fig. 3C). An increase in S4L was an index of an anticonvulsant effect, while microinjection of three doses of ramosetron had no significant effect on S5D (Fig. 3D).

### Effect of ramosetron and kindling on locomotion (in OFT) test

**Fig. 4:**
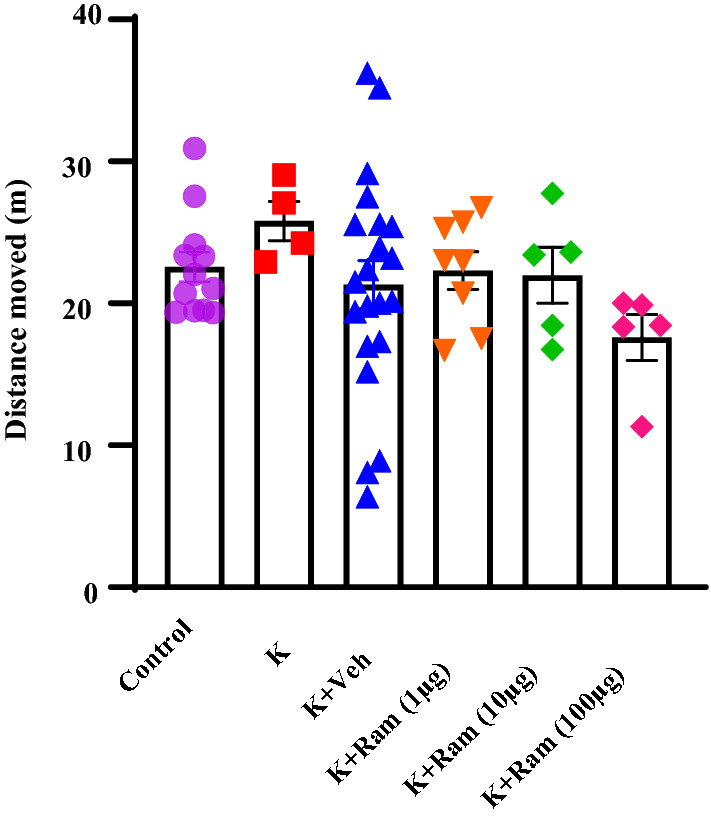
Effect of ramosetron and kindling on locomotion (in OFT) test. The experimental groups were found with no significant difference in locomotor activity. There were not significant differences in distance traveled among experimental groups. Data are expressed as mean ± SEM

The open-field test (OFT) measures locomotor activity, motor impairment, and anxiety in rodents. OFT was first developed to measure emotions in rats [33]. The experimental groups were found with no significant difference in locomotor activity. There were not significant differences in distance traveled [*P* = 0.4170, one-way ANOVA, Fig. 4] among experimental groups. Thus, these results confirmed that ramosetron and kindling do not affect locomotion.

### Effect of different doses of ramosetron on kindling-induced impairment in novel object-recognition test

**Fig. 5:**
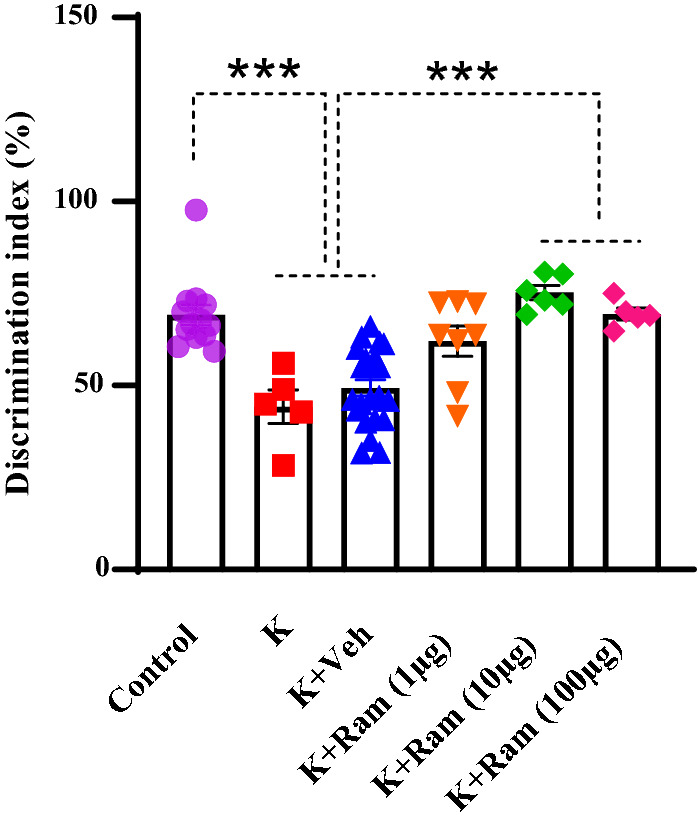
Effect of different doses of ramosetron on kindling-induced impairment in novel object-recognition test. Microinjection of ramosetron (10 and 100 μg/0.5 µl) increased the discrimination index. Data are expressed as mean ± SEM. ***P < 0.001

Considering the role of 5HT_3_ receptors in cognitive behaviors [23] and the role of EC in novel object recognition test [25], the probable effect of ramosetron on the probable effect of ramosetron on electrical amygdala kindling-induced memory impairment was also evaluated. NOR was first developed by Ennanceur and Delacour, according to the spontaneous behavior of animals for recognizing a novel object in a familiar environment [37]. Recognition memory as a type of declarative memory evaluates an animal’s capability for judging or discriminating between objects, considering visual and tactile data [37, 38]. In this work, the NOR test was used for evaluating the ramosetron effects on cognitive flexibility in a rat model of amygdala kindling. The 5-HT3 receptor antagonist, ramosetron, was microinjected into the EC of amygdala kindled animals (1, 10 or 100 µg/0.5 µl) 5 min before the NOR and seizure experiment. A one-way ANOVA followed by post hoc comparisons showed the memory restoring effect of this agent at 10 and 100 µg/0.5 µl (*P* < 0.001), but not at 1 µg/0.5 µl (*P* > 0.05; Fig. 5). Microinjection of ramosetron (10 and 100 μg/0.5 µl) increased the discrimination index in the kindled animals.

### Effect of different doses of ramosetron on kindling-induced impairment in spontaneous alternation in Y-maze test

**Fig. 6:**
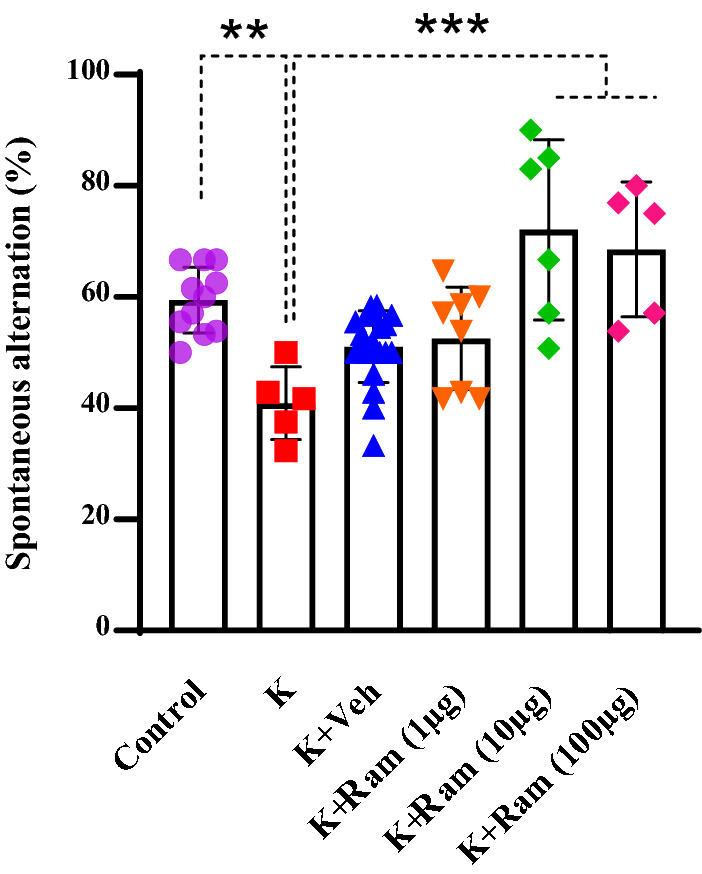
Effect of different doses of ramosetron on kindling-induced impairment in spontaneous alternation in Y-maze test. Microinjection of ramosetron (10 and 100 µg/0.5 µl) in fully kindled animals reversed the kindling induced changes in the percentage of spontaneous alternation. The Data are shown as mean ± SEM. **P < 0.01 and ***P < 0.001

Considering the role of 5HT_3_ receptors in cognitive behaviors [23] and the role of EC in working memory [24], the probable effect of ramosetron on the probable effect of ramosetron on electrical amygdala kindling-induced memory impairment was also evaluated. Y Maze spontaneous alternation is a behavioral test based on the animals' natural curiosity for exploration. One-way ANOVA analysis showed that there was a significant difference in the percentage of spontaneous alternation among the experimental groups (Fig. 6). Kindled animals showed significant decrease in the percentage of spontaneous alternation compared with control animals (*P* < 0.01). Administration of ramosetron (10 and 100 µg/0.5 µl) in fully kindled animals reversed the kindling induced changes in the percentage of spontaneous alternation (*P* < 0.001).

## Discussion

The principal goal of this study was to assay the role of EC’s 5-HT_3_ receptors on the severity of seizure and learning and memory impairments by electrical kindling of amygdala in rats. The results of the present study demonstrated that intra-EC microinjection of 5-HT_3_ receptor antagonist (ramosetron) reduced the amygdala kindled seizure severity, suggesting a role for EC’s 5-HT_3_ receptors in brain excitability and expression of convulsive discharges. It also showed that blockade of EC’s-5-HT_3_ receptors by the high dose of ramosetron (100 µg / 0.5 µl) reduced ADD and increased stage 4 latency in kindled rats. In addition the microinjection of ramosetron (10 and 100 µg / 0.5 µl) increased the discrimination index (in NOR test) and spontaneous alternation behavior (in Y-maze task) in fully kindled rats. Therefore, EC’s 5-HT_3_ receptors are involved in mediating the anticonvulsant effects of 5-HT in fully kindled animals and reduces learning and memory impairments. In addition, our results confirmed that ramosetron and kindling do not affect locomotion.

The EC is a major source of inputs to the hippocampus. The cingulate cortex, temporal lobe cortex, amygdala, orbital cortex, and olfactory bulb all have inputs to the hippocampus via the EC [7]. Previous experiments have shown that the EC has an important role in development of amygdaloid kindling [39] and plays a pivotal role in epileptogenesis and seizures. Another study has shown a prominent role of inhibitory networks in the EC during the transition to seizure [40]. In addition, electrical stimulation of EC interferes with the activity of specific areas of the brain, such as the amygdala, the piriform cortex, and the hippocampus [41]. Therefore, EC may be a promising target for intervention in epilepsy.

Previous studies have shown the role of serotonin as an important neurotransmitter in seizure development and epileptogenesis [3]. It has been postulated that a deduction in serotonergic neurotransmission may be the etiology of seizures experienced in a certain subset of epileptic patients [42, 43], in parallel, 5-HT depletion may intensify seizure in genetic models of epilepsy and chemically kindled animal [44, 45]. As mentioned in the introduction, the EC expresses high density of serotonergic receptors and especially 5-HT_3_ receptors [11]. Based on our results, selective antagonism of baseline 5-HT_3_ receptors activity in EC by high dose of ramosetron decreased the amygdala kindling-induced clonic seizures. Ramosetron showed high affinity for cloned human and rat 5-HT_3_ receptors, while its affinities for other receptors, transporters, ion channels, and enzymes were negligible, it means that obtained results is related to only the blocked of 5-HT_3_ receptors not others subtypes in EC [46].

Ramosetron is a recently developed selective 5-HT_3_ receptor antagonist. It exhibits significantly greater binding affinity for 5-HT_3_ receptors with a slower dissociation rate from receptor binding, resulting in more potent and longer receptor antagonizing effects compared with older 5-HT_3_ receptor antagonists [47, 48]. It was reported that ramosetron is more potent with a longer duration of action than granisetron in the prevention of emesis after cisplatin chemotherapy [49–51]. Treatment with the high-dose of ramosetron (100 μg/0.5 μl) 5 min before kindling stimulation reduced the kindled seizures parameters, i.e., ramosetron had anticonvulsant effect on kindling severity. In this regard, it has been reported that pharmacological stimulation of the serotonergic system applies no or slight enhancing effect, whereas pharmacological inhibition of this system modifies and postpones the amygdala kindled seizures in rabbits. It has been suggested that the role of 5-HT in the acquisition of kindling epileptogenesis differs depending on the 5-HT receptor subtypes [52].

Moreover, another study has revealed that m-chlorophenylbiguanide as an 5-HT_3_ receptor agonist, increases the duration of fully kindled seizures and facilitates the developmental seizure process which confirm the excitatory role of 5-HT_3_ receptors in the kindled animal [14]. In the same way there are other studies that indicated the anticonvulsant effects of 5-HT_3_ receptor antagonist as Semenova and Ticku have shown the decreases in the severity and increases the latency of audiogenic seizures in DBA/2 J mice [53]. It has been reported that 5-HT_3_ receptor antagonist increases the sensitivity to ethanol withdrawal seizures [54] and decreases the primary afterdischarge duration and the latency of secondary afterdischarge in hippocampal partial seizures generated by low-frequency electrical stimulation [55].

However, blockade of 5-HT_3_ receptors by the high dose of ramosetron reduces the electrophysiological parameter of ADD that is related to the activity of the temporal circuits in the registration area [56]. It seems that the ramosetron reduces this parameter during the kindled seizure procedure by inhibiting the neuronal circuits of the amygdala area. In the present study ramosetron prolongs the generalization stage of seizures in kindling. Given that S4L is the generalization rate index of the seizure attacks [56]; therefore, it is possible that ramosetron application may suppress the increased susceptibility to the second seizure. Other related studies exist in the literature (for example Taha and colleagues) which have demonstrated that enhancement of 5-HT_3_ receptor function results in as anticonvulsant effect in the PTZ induced seizure model and that selective antagonism at the 5-HT_3_ receptor yields proconvulsive effects [15]. It has also been reported that activation of 5-HT_3_ receptor by different agents increases the duration of fully-kindled seizures and facilitates the developmental seizure process in different models of kindling [1, 14, 15].

In addition, at the cellular level, postsynaptic 5-HT_3_ receptors have been shown to mediate fast excitatory synaptic transmission in rat neocortical interneurons, amygdala, and hippocampus [57–60]. 5-HT_3_ receptors are also present on presynaptic nerve ending. While there is some evidence for the role of 5-HT_3_ receptor in modulation of neurotransmitter release [61, 62], but they are inconclusive [63]. However, it has been shown that 5-HT_3_ receptor activation reduces the release of acetylcholine (ACh) in the EC [64]. In addition, ondansetron and granisetron as 5-HT_3_ receptor antagonists, in a concentration-dependent manner, increase ACh release in the entorhinal slices, while the use of 5-HT_3_ receptor agonists has no effect on ACh release but completely blocks the ondansetron-induced enhancement in ACh release [65]. These results suggest that activation of 5-HT_3_ receptors tonically inhibits ACh release in the EC. However, it has recently been reported that no significant decrease or increase in ACh release is observed with either the 5-HT_3_ receptor agonists or antagonists [66] casting doubts on the effects of 5-HT_3_ receptor activation on ACh release in the EC. Thus, the mechanisms of 5-HT_3_-mediated receptor inhibition in ACh release are unclear. Activation of muscarinic acetylcholine receptors (mAChRs) (for example by pilocarpine as an acetylcholine agonist) has been widely confirmed to induce seizures. Pilocarpine is also known to activate other mAChRs. These receptors could also play a role in generating and sustaining pilocarpine-induced seizures, contributing to the initiation of a seizure and its seriousness [67]. Based on above mentioned mechanism, the effects of ramosetron into EC to enhance severity of seizure can be explained as the effects of this drug on Ach release which the clear mechanisms remain to be elucidated.

Clinical studies have reported cognitive impairments in epileptic patients [68]. Serotonin plays an important role in emotional and motivational aspects of human behavior, including anxiety, depression, impulsivity, etc. Several clinically effective drugs work through 5-HT systems. Previous studies have suggested that these effects play an important role in learning and memory processes. The role of serotonin is related to memory and/or behavioral or emotional aspects although, the main question that remains is whether 5-HT receptor subtypes are directly or indirectly involved in the physiological basis of memory and/or pathogenicity of memory impairments? [69]. The findings of the current study showed that intra-EC microinjection of ramosetron restored cognitive impairments in kindled animals. EC and hippocampus has been shown to be essential for object recognition memory [70, 71]. Previous preclinical data have demonstrated that 5-HT_3_ receptor antagonists are able to improve memory in some preclinical cognitive dysfunction models [23, 72]. In addition to being innervated by serotonergic fibers, the EC also expresses high density of serotonergic receptors5-HT_3_ receptors are distributed in the EC, hippocampus CA1 area, amygdala, substantia nigra, and brainstem, and have strong expression [73].

The current results showed that the 5-HT_3_ receptors in the EC are involved in the novel object recognition test and spontaneous alternation behavior due to the fact that ramosetron increased the discrimination index and spontaneous alternation in fully kindled rats. It seems that ramosetron exerts its memory-enhancing effects through the manipulation of cholinergic [74] and glutamatergic neurotransmission [75], it has also displayed that activation of 5-HT_3_ receptors inhibits ACh release in the EC [64].

As the EC itself is known to be important for place recognition [76], it is really important to examine if ramosetron can improve memory formation independent of epilepsy. This is a limitation for our study that should be addressed in future studies. Although it has recently been shown that ondansetron, a specific 5HT_3_ receptor antagonist, can improve seizures and associated memory deficits in PTZ mice [77].

## Conclusion

In conclusion, the present study demonstrates an anticonvulsant role for a selective 5-HT_3_ receptor antagonist, suggesting an excitatory role of 5-HT_3_ receptors in the amygdala kindling model of epilepsy. Furthermore, this anticonvulsive effect was accompanied with a restoring effect in cognitive behavior in novel object recognition and Y-maze tests.

## Data Availability

All data and materials are available upon request.
